# Stochastic gene expression: bacterial elites in chemotaxis

**DOI:** 10.15252/msb.20167458

**Published:** 2017-01-23

**Authors:** Simon van Vliet, Martin Ackermann

**Affiliations:** ^1^ Department of Environmental Systems ScienceInstitute of Biogeochemistry and Pollutant DynamicsETH ZurichZurichSwitzerland; ^2^ Department of Environmental MicrobiologyEawagDübendorfSwitzerland

**Keywords:** Microbiology, Virology & Host Pathogen Interaction, Quantitative Biology & Dynamical Systems, Signal Transduction

## Abstract

Even in the absence of genetic or environmental differences, cells differ from each other in their molecular make‐up. The consequences of these phenotypic differences are often not well understood. New work by Waite *et al* (2016) directly links variation in the molecular composition of individual bacterial cells to their population‐level performance.

Bacteria are highly plastic: a single genotype can display a range of phenotypes in response to different environmental conditions. Strikingly, there can be phenotypic variation even in the absence of environmental variation due to the stochastic nature of gene expression (Raj & van Oudenaarden, 2008). As a result, cells in bacterial populations typically differ from each other in their molecular make‐up. The mechanisms leading to this phenotypic variation are relatively well studied. However, it is often less clear whether this variation is biologically relevant, because it is usually hard to measure what the *consequences* of phenotypic variation are for how well cells perform. In a recent article published in *Molecular Systems Biology,* Thierry Emonet and colleagues (Waite *et al*, 2016) overcame this difficulty by studying the chemotactic swimming behaviour of *Escherichia coli* bacteria. Chemotaxis in *E. coli* is an ideal model system to map a cell's phenotype to its performance: the molecular players and their interactions are characterized in great detail, and the swimming behaviour of cells can readily be measured using video microscopy. The authors made clever use of this well‐characterized system to link variation in protein levels to variation in motility at the single‐cell level. In turn, this variation in motility was linked to variation in chemotactic performance at the population level—that is to how fast a cell population can race uphill in a gradient of a chemoattractant. The authors were thus able to causally link cell‐to‐cell variation in molecular make‐up to variation in population performance, adding important insight into the biological relevance of phenotypic variation.

*Escherichia coli* swims using a run‐and‐tumble strategy: during a run, a cell swims in a straight line, while it stays in place and randomizes its direction during a tumble (Berg, 2004). By alternating between runs and tumbles, the cell moves in random walk. Chemotaxis allows cells to change the intervals between tumbles based on the change in chemoattractant concentration during a run. This gives rise to a biased random walk towards locations with higher concentrations of the attractant. The distance a cell travels is expected to depend on the fraction of time a cell spends tumbling (known as the tumble bias): the lower the tumble bias, the more time the cells spend swimming and the further it should travel. The authors observed exactly such a link between tumble bias and swimming performance: cells with a lower tumble bias show a stronger performance and progress faster through a chemoattractant gradient. Strikingly, this relationship is non‐linear: the cells with the lowest tumble bias have a much higher performance than all other cells.

In an earlier paper (Dufour *et al*, 2016), the authors established a link between the variation in the molecular composition of a cell's chemotactic network and its tumble bias. Using mutant strains with tunable expression levels of several chemotaxis proteins, the authors could now extend this finding and directly link the variation in protein levels to the variation in swimming performance. Changes in protein levels that lead to a lower tumble bias increase the speed with which cells climb the attractant gradient, while a mutant that does not tumble at all performs very poorly. Combining all data, the authors can thus causally link variation in molecular composition to variation in performance.

Due to the non‐linear mapping from phenotype to performance, the overall performance of the whole group is strongly dependent on a small fraction of individuals whose performance sticks out from everybody else. Because of this, the performance of the population can no longer be simply predicted based on the mean phenotype of the population. In other words, the average swimming performance of the population is larger than the performance of cells with an average tumbling phenotype (Fig 1). It is therefore necessary to understand the distribution of the phenotypes and performance of individual cells to describe the overall performance of the population.

**Figure 1:**
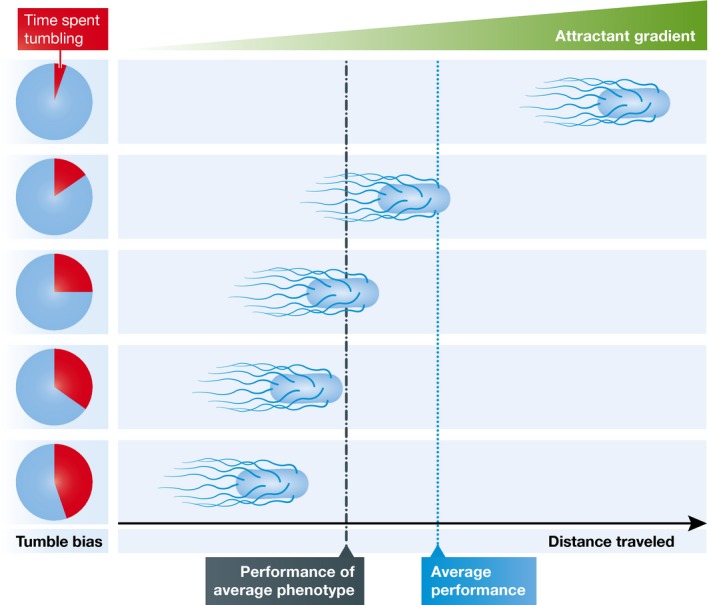
Rare phenotypes can dramatically improve population performance A population consists of cells that differ in their tumble bias (pie charts on the left). The mapping between tumble bias and distance travelled is non‐linear: cells with a low tumble bias travel much further up a gradient in chemoattractant. Due to this non‐linearity, the average performance (blue dashed line) is higher than the performance of the average phenotype (dark grey dashed line).

A deep insight resulting from this analysis is that cell‐to‐cell variation should not obligatorily been interpreted as mere “noise” but rather as a way to modulate the *shape* of the distribution of a cellular phenotype, which will ultimately determine the population‐level functional performance, with potentially non‐intuitive consequences.

This work raises an important question: why did natural selection not fine‐tune this bacterium's gene regulatory network so that most individuals have a low tumble bias and a high performance? While the scarcity of cells with low tumble bias could be a consequence of laboratory cultivation (Waite *et al*, 2016), we see three additional possible explanations. First, it is possible that there are molecular constraints that prevent this outcome. Finding such constraints would offer intriguing insights into the limitations of cellular control. Second, it is possible that this majority of underachievers performs well in other tasks that are important in the life of *E. coli*. The distribution of phenotypes could thus be part of a bet‐hedging strategy where different groups of cells are adapted to different environmental conditions. Finally, differences in chemotactic performance do not necessarily translate to differences in reproductive success. There would be no selection for improved swimming performance in environments where the arrival time at the source of the attractant does not translate into a reproductive benefit.

While chemotactic and reproductive performance could indeed be decoupled in some environments, the opposite is also conceivable: it is possible that a small group of individuals with the highest chemotactic performance has access to ephemeral hot spots of nutrients that other individuals cannot reach in due time (Stocker, 2012). In this case, the chemotactic elite that Waite and colleagues discovered could strongly affect how bacteria live and grow in spatially and temporally dynamic environments (Frankel *et al*, 2014). This highlights an important challenge in matching phenotypic variation to differences in reproductive performance: we need to understand the challenges that individuals face in their natural environment to fully understand the selective pressures operating on the distributions of phenotypes. Future work could address these questions using microfluidic devices mimicking situations encountered in the natural environment of these bacteria.
